# Physical and psychological differences between athletes with and without chronic primary low back pain: a scoping review

**DOI:** 10.3389/fspor.2025.1621796

**Published:** 2025-08-04

**Authors:** Clément Prunault, Guillaume Vadez, Martin Descarreaux, Jacques Abboud

**Affiliations:** ^1^ Department of Anatomy, Université du Québec à Trois-Rivières, Trois-Rivières, QC, Canada; ^2^ Research Group on Neuromusculoskeletal Disorders (GRAN), Trois-Rivières, QC, Canada; ^3^ Department of Human Kinetics, Université du Québec à Trois-Rivières, Trois-Rivières, QC, Canada

**Keywords:** chronic low back pain, athletes, electromyograhy (EMG), kinematic, psychology, kinetic

## Abstract

**Objective:**

Low back pain among athletes varies by sport, age, and sex, affecting performance and contributing to sports retirement. Recently, there has been an increasing focus on chronic primary low back pain (CPLBP) in athletes. The aim of this scoping review is to examine the existing literature on CPLBP, focusing on the physical and psychological differences between athletes with and without CPLBP.

**Design and Methods:**

A systematic search across MEDLINE, CINAHL, SportDiscus, and PsycINFO, guided by the Preferred Reporting Items for Systematic Reviews and Meta-Analyses Guidelines (PRISMA), identified 11 relevant studies. Three key concepts guided the search: “chronic low back pain”, “physical and psychological characteristics”, and “athletes”.

**Results:**

From 1,767 screened articles, 11 studies involving 717 athletes (301 with CPLBP and 416 without) met the inclusion criteria. Most studies showed that athletes with CPLBP had significantly lower trunk muscle activation amplitudes and delayed onset. One study found that athletes with CPLBP had significantly reduced trunk extension strength. Kinematic evaluations showed a significant increase in trunk damping and lumbar extension in athletes with CPLBP compared to athletes without CPLBP. However, other studies reported no significant differences in trunk muscle activity, kinetic and kinematic variables. Finally, athletes with CPLBP reported significantly higher kinesiophobia, anxiety and pain catastrophizing scores.

**Conclusions:**

This review shows significant physiological and psychological differences between athletes with and without CPLBP. Future research should focus on sport-specific assessments of CPLBP and its impact on performance.

## Introduction

Low back pain (LBP) is defined as pain typically located between the lower rib margins and the buttock (1). It may be accompanied by additional pain in one or both legs (1). It is the most common musculoskeletal complaint worldwide and the leading cause of years lived with disability (2). LBP affects up to 84% of individuals in the general population during their lifetime (3). In most individuals with LBP, the exact nociceptive source often cannot be identified, consequently classifying the condition as primary LBP, formerly known as “nonspecific LBP” (4). The International Association for the Study of Pain defines chronic primary low back pain (CPLBP) as pain that persists or recurs for longer than three months, and is associated with emotional distress or functional disability, and symptoms that cannot be better accounted for by another diagnosis, such as tissue damage or a disease process (5).

LBP risk factors are numerous, including genetic factors, female sex, lifestyle factors such as sedentary behaviours, obesity, smoking, psychosocial factors, poor coping mechanisms and occupational hazards (6). Importantly, these factors may also contribute to the chronicity of LBP (6). CPLBP results from complex interactions between biological, psychological, and social factors (7). For instance, fear avoidance beliefs, depression, anxiety, catastrophic thinking, and familial and social stress are highly prevalent in adults with CPLBP (1). In addition, individuals with CPLBP show alterations in neuromuscular function, such as delayed muscle activation, particularly in the erector spinae during perturbations (8). Furthermore, a decrease in muscle strength and endurance of back muscles is observed in individuals with CPLBP compared to those without CPLBP (8).

In athletes, current evidence indicates that the lifetime prevalence of LBP significantly varies across studies, ranging from 1%–94% and is influenced by factors such as sport type, age, and gender, with a general trend of increasing prevalence with age (9–12). For example, the highest point prevalences of LBP in athletes were found among skiers (13), floorball players (14) and rowers (15). Although the prevalence of CPLBP across different sports remains unclear (12), a recent study reported an overall prevalence of 14.1% among university athletes from six disciplines (16).

Certain risk factors for LBP in the general population, such as psychological factors, have been described among athletes as well (16). However, other factors commonly associated with LBP, like decreased physical activity, smoking, and obesity, are less likely to be investigated in athletes due to their physically active lifestyle (17). Psychological factors such as stress, fatigue, anxiety, impaired sleep and mood have been identified as risk factors for athletes (18, 19). Moreover, specific types of sporting activities may also act as risk factors, including regular back hyperextension (15), aberrant movements, strength deficits, suboptimal movements (20), bending and twisting, frequent lifting, or intense workloads (12). Practicing an endurance sport may also represent an additional risk factor, as a high prevalence of LBP has been reported among cross-country skiers and rowers (21). In addition, the intensive practice of sports induces physical and psychological stress, which may contribute to the development of CPLBP in athletes (22). Although extensive information, including epidemiological data and risk factors, is available in individuals with CPLBP, authors highlighted the need for high-quality studies reporting LBP in terms of its chronicity specifically in athletes (16). To this day, no comprehensive synthesis of physical and psychological differences between athletes with and without CPLBP currently exists.

This scoping review aims to examine the existing literature on CPLBP in athletes, with a particular focus on the physical and psychological differences between athletes with and without CPLBP. By addressing this objective, the review aims to provide a comprehensive overview of the current knowledge and gaps in understanding the complex nature of CPLBP in athletes.

## Methods

This scoping review is reported in accordance with the PRISMA-ScR (Preferred Reporting Items for Systematic Reviews and Meta-Analyses extension for Scoping Reviews) guidelines (23). Scoping reviews are designed to inform broad research questions, such as the impact of CPLBP-related physiological and psychological characteristics on athletes, and to refine subsequent research inquiries (24).

### Protocol

No protocol was registered for this scoping review.

### Identifying the research question

The objective of this scoping review was to address the following research question: What are the physical and psychological differences between athletes with and without CPLBP?.

In this review, physical differences refer specifically to neuromuscular and biomechanical outcomes, including muscle activity (e.g., EMG), kinetic measures (e.g., ground reaction forces), and kinematic measures (e.g., joint angles, movement patterns). Psychological differences refer to cognitive-emotional factors like pain catastrophizing, kinesiophobia, fear-avoidance beliefs, anxiety, stress, coping strategies, generally assessed through self-report questionnaires. The detailed list of physical and psychological outcomes and assessment tools can be found in Supplementary Appendix 2.

### Study selection and screening

#### Inclusion and exclusion criteria

To be included, all studies had to be published in a peer-reviewed journal and written in either French or English. Eligible studies had to investigate trained athletes with CPLBP and include a comparison group of athletes without CPLBP. Only studies reporting comparisons on muscle activity, kinetic, kinematic, or psychological outcomes were included. For the purpose of this scoping review, athlete status was defined based on McKay et al. (2022) (25) classification, with included participants required to meet at least Tier 2 criteria, corresponding to trained athletes. Athletes classified as Tier 3 or higher were also included, if they met the minimum Tier 2 definition. The athletes had to be identified with a specific sport, and details such as the number of training sessions per week, level of competition, or affiliation with a university team were required for the inclusion of a given study. Studies were excluded whenever it was unclear whether the athletes corresponded to the “trained” group of athletes since we wanted to exclude recreational athletes from the scoping review. Moreover, only studies that clearly defined CPLBP as pain that persists or recurs for longer than three months were selected (5). The following types of publications were excluded: opinion and commentary papers, letters, editor responses, conference abstracts, and study protocols. Additionally, reviews, meta-analyses and randomized control trial studies were excluded, as only primary studies that compared athletes with and without CPLBP were searched. Studies involving athletes younger than 18 years of age were also excluded.

### Identifying relevant studies

The article search was conducted with the assistance of a librarian at the University of Quebec in Trois-Rivières from February 2024 up to March 2024 in the following databases: MEDLINE, CINAHL, SportDiscus and PsycINFO. Terms (MESH or non-MESH) have been used in combination and adapted to suit the database, appearing in the title and abstract. The three main concepts were low back pain, athlete, and the physical and psychological characteristics. All keywords by concept are listed in Supplementary Appendix 1. Additional data sources were searched, including the authors' pre-existing knowledge of the literature and the reference lists of articles, with the aim of identifying other relevant published peer-reviewed studies. An EndNote library, version 21.2 (Clarivate Analytics, Philadelphia, PA, United States), was created to import all studies from the initial search results and identify duplicates. Next, a review using Rayyan software (https://www.rayyan.ai/, Cambridge, MA, United States) was conducted to identify additional duplicates and screen the articles. The search strategy was not restricted by years of publication.

### Study screening

The article search was conducted by one of the authors (C.P.), whereas the screening of records by title and abstract was performed by two independent reviewers (C.P. and G.V.). In case of disagreements between the two reviewers, further review was conducted by J.A. and M.D. The full texts of the studies deemed relevant and potentially relevant were then assessed by two reviewers (C.P. and G.V.) to determine the final set of eligible studies for this scoping review.

### Charting the data

To extract relevant data and information from the eligible studies, a data extraction table was created in Excel, which included author and year of publication, country, study objectives, study design, pathology, population (level of athlete, sport, age), type of evaluation (e.g., cardiovascular or reaction time task), variables (e.g., muscle activity, angle of movement), the tools used (e.g., electromyography or kinematic system) and main findings regarding physical or psychological differences between athletes with and without CPLBP for each study. The extraction of all information was conducted by one reviewer (C.P.) and double-checked by two other reviewers (J.A. and M.D.).

### Synthesis of results

The results from the included studies were synthesized descriptively. Before presenting the main findings, studies were first grouped based on the evaluation protocols and the tools used. This process made it easier to identify commonalities and differences between the studies.

Moreover, the main findings were classified into four categories based on the outcome measures reported in each study: electromyographic differences, kinetic differences, kinematic differences and psychological differences between athletes with and without CPLBP. This categorisation allowed a targeted comparison of physical and psychological differences between athletes with and without CPLBP.

## Results

**Figure 1:**
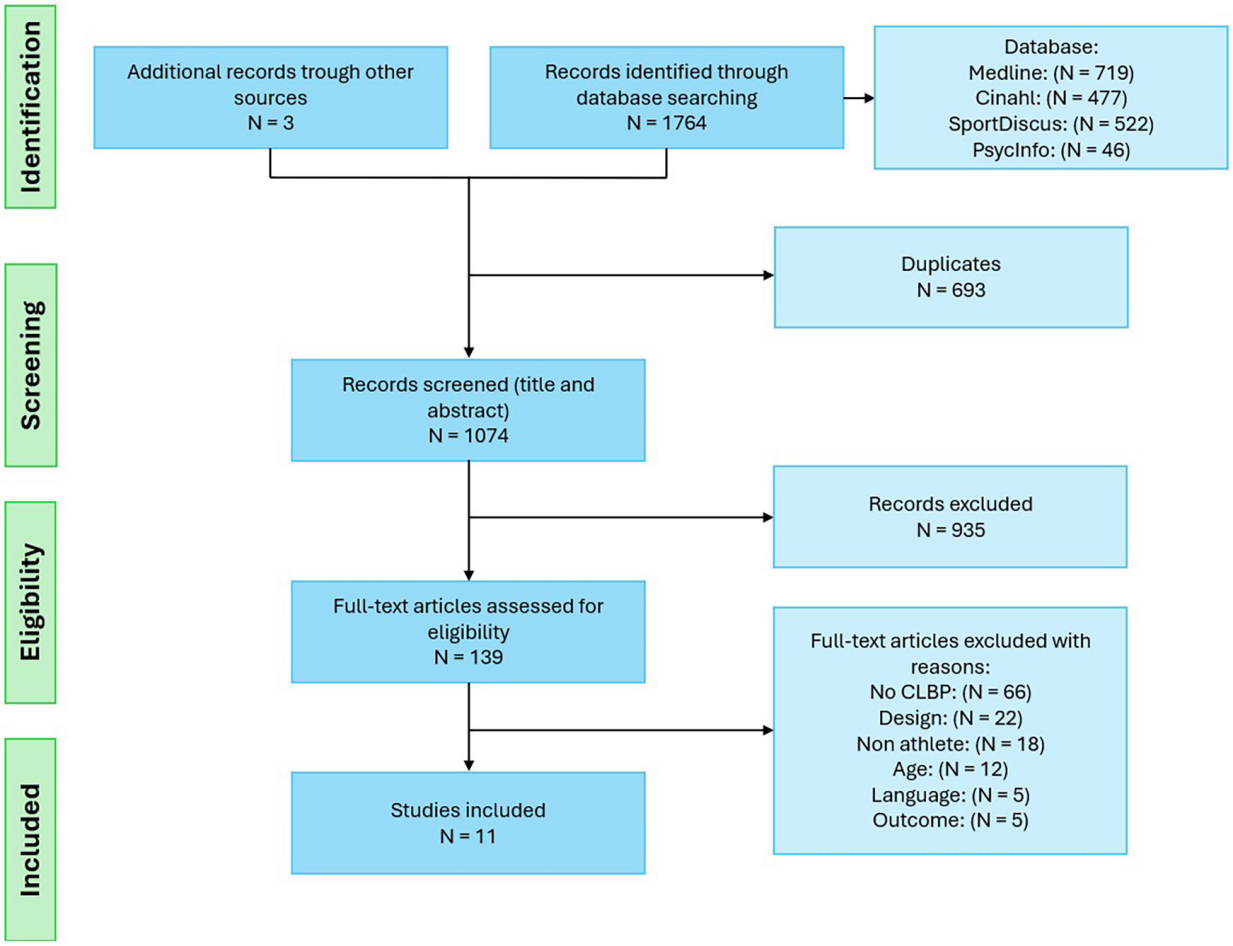
Flow diagram of study selection.

A total of 1,767 articles were identified, from which 11 fulfilled the inclusion criteria (refer to Figure 1). Three references were identified through searches on the website: connected papers (https://www.connectedpapers.com/, San Francisco, CA, United States). A PRISMA-ScR diagram of the search results, including the reasons for exclusion, is shown in Figure 1. Of the 11 studies, eight were cross-sectional studies (26–33), one was an experimental longitudinal study (34), one was a case-control study (35), and one was a pilot study (36). The selected studies were conducted in Iran (26, 29, 31, 35), Germany (28, 34), India (32, 33), Canada (27), Australia (36), and Japan (30). In total, 717 trained athletes are included in this scoping review (301 athletes with CPLBP and 416 athletes without CPLBP), with 449 men and 268 women. The mean age of athletes with CPLBP is 27 ± 5 years, while the mean age of athletes without CPLBP is 26 ± 5 years.

**Figure 2:**
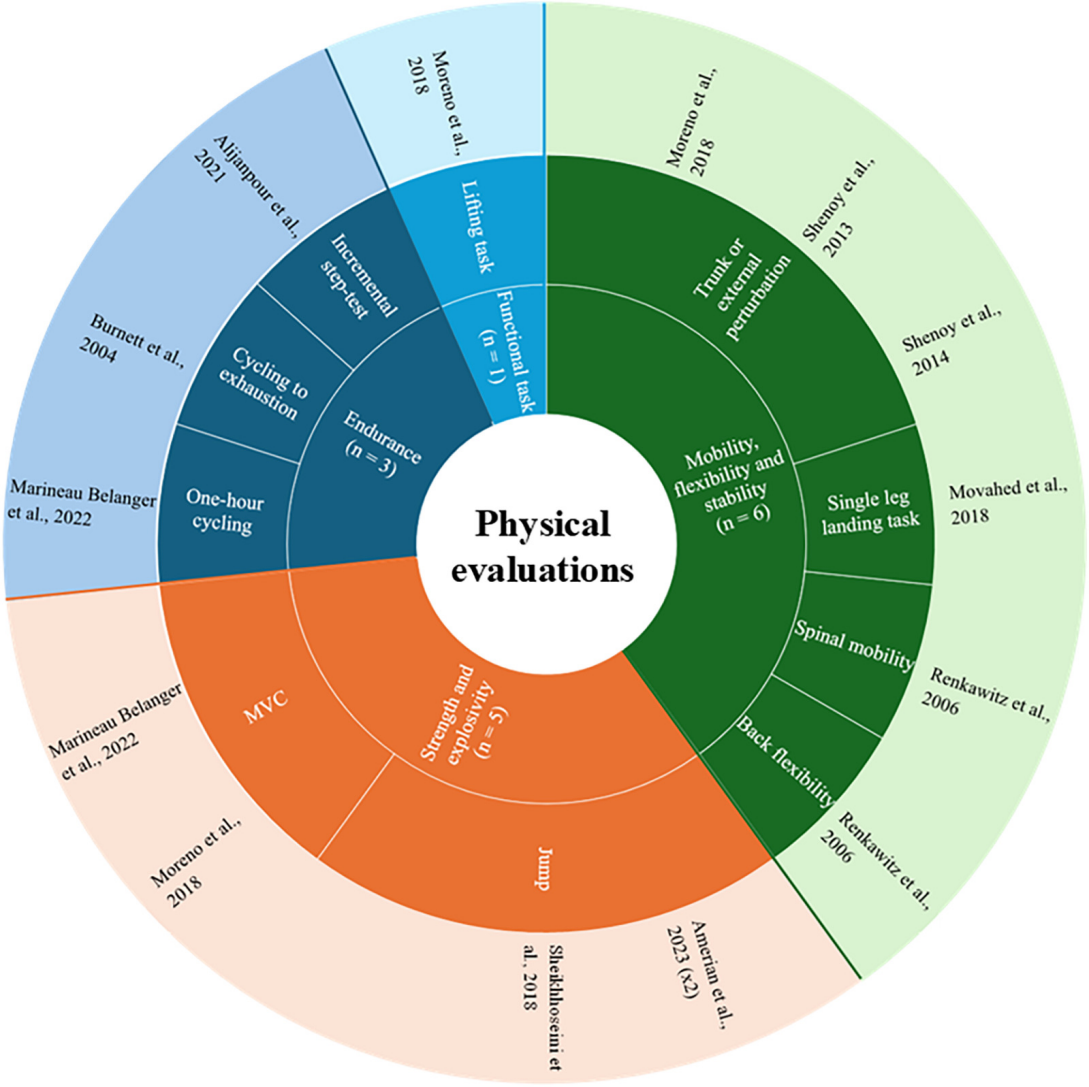
Summary of the different types of evaluations present in the included studies.

Among the evaluations conducted in the studies, the majority are mobility, flexibility, and stability evaluations (Figure 2). Strength and explosiveness evaluations are also commonly featured in these studies. Table 1 summarizes the sports associated with these evaluations in the included studies.

**Table 1:** The association between sports and physical evaluations.

Physical evaluations	Sports	References
Endurance	Rowing, Cycling	Alijanpour et al., (26); Burnett et al., (36); Marineau Belanger et al., (27)
Strength and explosivity	Cycling, Jumping, Cutting, Pivoting Sports, Handball, Judo, Gymnastics, Athletics	Amerian et al., (35); Marineau Belanger et al., (27); Moreno et al., (28); Sheikhhoseini et al., (31)
Mobility, flexibility and stability	Tennis, Soccer, Hockey, Basketball, Handball, Judo, Gymnastics, Athletics, Volleyball	Moreno et al., (28); Movahed et al., (29); Renkawitz et al., (34); Shenoy et al., (32); (33)
Functional task	Soccer, Handball, Judo, Gymnastics, Athletics	Moreno et al., (28)

### Tools to measure physical and psychological characteristics

In these 11 studies, six used electromyography (EMG) (27, 28, 32–34, 36), four used kinetic systems (27, 28, 34, 35), such as force plate or dynamometer, and six used kinematic systems (26–29, 31, 36). Two used active systems (27, 36) and four used passive system (26, 28, 29, 31). Renkawitz et al. (2006) (34) conducted clinical evaluations assessing spinal mobility and back flexibility.

Regarding self-reported outcomes to compare athletes with and without CPLBP, one study used a visual analog scale to assess back pain during cycling endurance exercise (27). Other studies used self-reported outcomes to assess kinesiophobia (Tampa Scale for Kinesiophobia) (30), fear-avoidance (Fear-Avoidance Beliefs Questionnaire-Physical Activity subscale and Athlete Fear Avoidance Questionnaire) (30), catastrophizing (Pain Catastrophizing Scale) (30), anxiety (Pain Anxiety Symptom Scale-20 and State-Trait Anxiety Inventory questionnaire) (35), and perceived exertion (Borg scale) (27).

### Synthesis of data

#### Physical differences

*Muscle activity:* Athletes with CPLBP exhibit significantly lower EMG amplitudes in the rectus abdominis and erector spinae muscles compared to athletes without CPLBP during unexpected tasks, while only in the rectus abdominis during expected tasks (32). In tasks where athletes were instructed to either “let go” or “resist” trunk movements in response to perturbations, athletes with CPLBP also showed significantly reduced EMG amplitudes for the rectus abdominis, and additionally for the erector spinae during the “resist” command compared to athletes without CPLBP (33). Conversely, other studies (27, 36) found no significant differences in trunk muscle activation between athletes with and without CPLBP (Table 2).

**Table 2:** Significant and nonsignificant differences in muscle activity between athletes with and without CPLBP**.**

Physical variable	Evaluation	Muscles		*Authors*
Muscle activity	Unexpected task	ES delay (NA)	RA delay (NA)	*Shenoy* et al.*,* (32)
	Expected task	ES delay	RA delay	*Shenoy* et al.*,* (32)
	Sudden perturbations	Lumbar ES delay (*p* = 0.019), MD (7 ms); Thoracic ES delay (*p* = 0.025) MD (5 ms)		*Moreno Catalá* et al.*,* (28)
	Unexpected task	ES RMS (*p* = 0.05), MD (25,56 µV)	RA RMS (*p* = 0.05), MD (35.50 µV)	*Shenoy* et al.*,* (32)
	Expected task	ES RMS	RA RMS (*p* = 0.05), MD (15.79 µV)	*Shenoy* et al.*,* (32)
	"Let go” command	ES RMS	RA RMS (*p* = 0.05), MD (15.76 µV)	*Shenoy* et al.*,* (33)
	"Resist” command	ES RMS (*p* = 0.05), MD (38.18 µV)	RA RMS (*p* = 0.05), MD (20.46 µV)	*Shenoy* et al.*,* (33)
	Cycling	ES RMS	Abdominal RMS	*Burnett* et al.*,* (36)
	Cycling	ES RMS		*Marineau Belanger* et al.*,* (27)
	Isometric trunk extension	Neuromuscular imbalance (between right and left ES) (*p* < 0.01), MD (22 IEMG quotients)		*Renkawitz* et al.*,* (34)

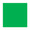
: Athletes with CPLBP showing more…, 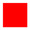: Athletes with CPLBP showing less…, 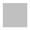: No difference between the two groups.

ES, erector spinae; RA, rectus abdominis; RMS, root mean square; MD, mean difference between athletes with and without CPLBP; NA, not applicable; p, *p* value; ms, milliseconds; µV, microvolts; IEMG, rectified integrated electromyography.

Regarding reaction time, athletes with CPLBP exhibited a significant delay in muscle activation onset, in the rectus abdominus and erector spinae muscles during unexpected tasks compared to athletes without CPLBP, but this delay was not observed during expected tasks (32). In contrast, another study indicated that athletes with CPLBP displayed significant shorter muscle reaction times after sudden perturbations, compared to athletes without CPLBP (28). To finish, athletes with CPLBP had significant higher neuromuscular imbalance between the right and left erector spinae at the L2 and L4 lumbar levels during maximum voluntary trunk extension contraction compared to athletes without CPLBP (34) (Table 2).

*Kinetic:* Trunk muscle strength was measured in four studies. One study demonstrated that athletes with CPLBP exhibit significantly less strength during trunk extension (not in flexion) compared to athletes without CPLBP (28). In contrast, other studies reported no significant difference between athletes with and without CPLBP (27, 34). Additionally, a study (35) found that during a double-leg vertical drop jump, CPLBP athletes with high pain-related anxiety exhibited significantly longer time to stabilization in all directions compared to athletes without CPLBP. Athletes without CPLBP also showed significantly greater oscillations in body balance distance during double-leg vertical drop jump than the CPLBP group. However, no significant differences in time to stabilization and postural oscillations were observed between athletes with and without CPLBP during a single-leg vertical jump (35) (Table 3).

**Table 3:** Significant and nonsignificant differences in kinetic and kinematic between athletes with and without CPLBP.

Physical variable	Evaluation	Variables				*Authors*
Kinetic	MVC	Strength in extension (*p* = 0.013–0.023), MD (0.4–0.8 Nm/kg)		Strength in flexion		*Moreno Catalá* et al.*,* (28)
	MVC	Strength				*Marineau Belanger* et al.*,* (27)
	MVC	Strength				*Renkawitz* et al.*,* (34)
	DVJ	Time to stabilization (*p* *=* 0.00), MD (0.55 s)		Oscillations (*p* = 0.05), MD (18.22 mm) and (*p* = 0.02), MD (20.25 mm) *		*Amerian* et al.*, 2023*
	SVJ	Time to stabilization		Oscillations		*Amerian* et al.*, 2023*
Kinematic	Sudden perturbations	Trunk damping (*p* = 0.018)		Trunk stiffness		*Moreno Catalá* et al.*,* (28)
	Single leg landing task	Lumbar extension initial contact (*p* = 0.025), MD (4.45°) GRF (*p* = 0.020), MD (−4.49°)		Other angles		*Movahed* et al.*,* (29)
	Rowing	Lower back ROM (*p* = 0.019), MD (4.38°)	Coordination (*p* < 0.05)	Coordination variability	Segment's ROM	*Alijanpour* et al.*,* (26)
	Cycling	Spinal flexion thoracic (*p* = 0.03)		Lumbar flexion angle		*Marineau Belanger* et al.*,* (27)
	Cycling	Lumbar flexion angle				*Burnett* et al.*,* (36)
	Jump-landing jump	Knee flexion (*p* = 0.029), MD (4.56°)	Hip flexion (*p* = 0.016), MD (10.74°)	Other angles		*Sheikhhoseini* et al.*,* (31)

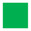
: Athletes with CPLBP showing more…, 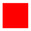: Athletes with CPLBP showing less…, 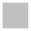: No difference between 2 groups.

MVC, maximum voluntary contraction; ROM, range of motion; DVJ, double-leg vertical drop jump; SVJ, single-leg vertical drop Jump; MD, mean difference between athletes with and without CPLBP; *p*, *p* value; Nm/kg, Newton-meters per kilogram; s, seconds; mm, millimeters; °, degrees.

*Kinematic:* Six studies evaluated the kinematic differences between athletes with and without CPLBP. Significant differences were observed between athletes with and without CPLBP including higher trunk damping (28), higher lumbar extension (29), and a higher lower back range of motion for athletes with CPLBP (26). However, no significant difference was found in trunk stiffness between athletes with and without CPLBP (28). During an incremental evaluation, rowers with CPLBP exhibited a significant decrease in coordination (lower trunk/lower back) in the sagittal plane during the early recovery and blade extraction phases compared to rowers without CPLBP (26). However, despite an opposing trend observed in rowers with CPLBP, there was no significant difference in coordination variability between rowers with and without CPLBP (26). During an endurance effort, cyclists suffering from CPLBP showed significantly less thoracic spinal flexion compared to the control cyclists (27). Finally, during jump-landing jump evaluations, athletes with CPLBP exhibited significantly less knee flexion and significantly greater hip flexion than athletes without CPLBP at the lowest point of the jump-land-jump phase (31). Despite these specific alterations, the studies included in the scoping review indicate that several aspects of movement remain similar between athletes with and without CPLBP, such as lumbar flexion angle (27, 36), segment's range of motion (ROM) (26), and other kinematic angles (29, 31) (Table 3).

#### Psychological differences

Only three studies compared athletes with and without CPLBP on psychological characteristics (27, 30, 35). Among these studies, two reported significant psychological differences between groups. Athletes with CPLBP showed significantly higher scores in anxiety (State-Trait Anxiety Inventory questionnaire-STAI) (35), fear-avoidance beliefs (Fear-Avoidance Beliefs Questionnaire physical activity-FABQ-PA), kinesiophobia (Tampa Scale for Kinesiophobia-TSK-11), and pain catastrophizing (Pain Catastrophizing Scale-PCS) (30). However, no significant difference was found on the Athlete Fear Avoidance Questionnaire (AFAQ), which is specifically designed for athletes (30). In addition, no difference between athletes with and without CPLBP was observed in perceived exertion during a cycling endurance task, as measured by the Borg scale (27).

## Discussion

The objective of this scoping review was to synthesize the current evidence regarding the physiological and psychological differences between athletes with CPLBP and athletes without CPLBP. Only 11 studies were included in this review, however, there has been a growth in research on this topic in recent years (37). The results revealed significant differences between athletes with and without CPLBP in terms of various physical, and psychological characteristics. Specifically, athletes with CPLBP seems to exhibit deficits in muscle activity (32–34), strength (28), and movement (26–29, 31, 35), as well as higher scores for kinesiophobia, pain catastrophizing, pain-related fear (30) and anxiety (35). These psychological factors can exacerbate pain and contribute to the chronicity of LBP (38), which could impact both the short-term and long-term trajectories of these athletes' careers. Nevertheless, the results remain highly variable, and several conflicting findings were identified in the selected studies.

For instance, the faster muscle reaction times observed in athletes with CPLBP after sudden perturbations (28), suggest that their muscles respond more quickly to sudden changes. However, another study, reported a delay in muscle activation latency of trunk muscles during unexpected perturbations in athletes with CPLBP (32). A systematic review by Abboud et al. (2017) (39) demonstrated a longer reflex latency for the erector spinae muscles in patients suffering from CPLBP compared to healthy participants and the study by Moreno Catalá et al. (2018) (28) appears to be the only one showing a shorter reaction time in individuals with CPLBP.

Studies by Shenoy et al. (2013, 2014) (32, 33) have shown that athletes with CPLBP often exhibit lower trunk muscle EMG amplitudes during unexpected or expected tasks, which is similar to observations made in individuals with CPLBP (39). Other studies, however, have shown opposite results, suggesting no differences in EMG amplitude between athletes with and without CPLBP (27, 36). This inconsistency may be attributed to the diversity of experimental protocols and the motor tasks evaluated. Hodges & Danneels (2019) (40) highlighted task-specific changes observed through EMG in the multifidus and erector spinae muscles during various tasks in individuals with CPLBP. These findings emphasize the importance of exploring the differences in neuromuscular control between athletes with and without CPLBP, as the variability in experimental protocols and task-specific muscle activation may account for the inconsistencies observed in the literature. Moreover, the variability of results can also be explained by interindividual variability. Interindividual variability seems to arise from the versatility of the complex trunk muscle system to enhance spinal protection in response to pain, using strategies specific to each individual (40), as predicted by contemporary models of pain adaptation (41). It seems that the variability in EMG measurements among athletes with CPLBP compared to athletes without CPLBP reflects a dynamic and personalized adaptation of the muscular system.

Findings regarding kinetics were also mixed. One study demonstrated a reduction in trunk extension strength in athletes with CPLBP (28). However, others studies have found no significant difference (27, 34). The review by Steele et al. (2014) (42) concluded that non-athletic individuals with CPLBP had decreased lumbar extensor strength. Such differences can be explained by the specific characteristics of the athletes studied. Indeed, despite the presence of chronic pain, similar strength levels in athletes with and without CPLBP may be due to their intensive training and daily routines. A systematic review has shown that regular training can mitigate the effects of CPLBP on muscle strength (43). Therefore, variations in the results may reflect neuromuscular adaptations specific to the sports and types of training, regardless of pain status.

Amerian et al. (2023) (35) demonstrated that athletes with CPLBP modify their postural control strategy, exhibiting significantly less oscillation compared to athletes without CPLBP during jump landing evaluations. According to new pain adaptation theory, adaptation to pain modifies mechanical behaviour such as altering movement and increasing stiffness as a protective response against further pain or injury, or the threat of either (41). According to this theory, athletes with CPLBP may adopt a protective strategy during jump landings, which could explain the significantly reduced oscillation observed compared to athletes without back pain. Future research should combine EMG measurements of trunk muscles with jump evaluations on a force platform to validate and understand the strategies adopted by athletes with CPLBP.

Furthermore, time to stabilization after double-leg vertical drop jump (35) was significantly longer in athletes with CPLBP than in those without CPLBP. Previous studies have shown alterations in neuromuscular control and proprioception deficits in the lumbopelvic region among individuals suffering from LBP, which could impact trunk postural control (44, 45). Recovery from instability during the sudden transition from a dynamic to a static state upon landing requires precise neuromuscular control of the trunk, which is impaired in individuals with LBP (44).

Regarding kinematic assessments, athletes with CPLBP exhibit significant alterations, such as increased lumbar extension (29) and an inability to adjust their coordination as exercise intensity increases (26). However, it is important to note that several aspects of postural control and movement remain similar between athletes with and without CPLBP, suggesting that certain motor abilities may not be consistently affected by CPLBP.

Nevertheless, a literature review shows that studies involving athletes with LBP have shown that they exhibit various motor control impairments, including alterations in kinematics, kinetics, muscle activation, and strength in the trunk (46).

Psychologically, athletes with CPLBP display significantly higher scores in kinesiophobia, catastrophizing, avoidance behaviours (30) and anxiety (35) than athletes without CPLBP. These psychological factors can exacerbate pain and contribute to the chronicity of LBP (6). The persistence of pain, despite a perceived effort similar to that of athletes without CPLBP (27), may indicate the complex influence of psychological factors on performance and pain perception in athletes with CPLBP. Based on the fear-avoidance model by Vlaeyen & Linton (2000) (47), kinesiophobia, catastrophizing, and avoidance behaviours are similarly elevated in the general population with CPLBP.

Beyond the psychological factors identified in this review (such as pain catastrophizing, kinesiophobia, fear-avoidance, and anxiety), a significant gap remains in the literature regarding other psychological factors particularly relevant to athletes with CPLBP such as mental stress, recovery-stress imbalance, cognitive and emotional endurance responses to pain, coping strategies, and mental fatigue. These factors were highlighted by Heidari et al. (2016) (22), who showed that both physical and psychological stress are associated to LBP chronicity in athletes. Kleinert et al. (2018) (48), also highlighted the need to consider stress-related psychological mechanisms in both LBP prevention and intervention. Our review did not identify any comparative studies that examined recovery-stress balance, mental fatigue, or coping strategies between athletes with and without CPLBP.

The physical and psychological differences observed between athletes with and without CPLBP could reflect causes or consequences of pain. Existing models and data suggest that pain can induce changes in motor control, such as altered muscle recruitment and movement patterns, which are initially protective mechanisms that may become maladaptive if maintained over time, potentially contributing to pain persistence (41). In athletes, such compensatory strategies may temporarily reduce spinal load but ultimately impair neuromuscular function. Similarly, reduced strength (28) or coordination (26) might result from decreased training due to pain but could also increase the risk of recurrence or injury.

Psychologically, Vlaeyen and Linton's (2000) (47) fear-avoidance model describes how pain-related fear, catastrophizing, and avoidance behaviors may initially develop in response to pain but can ultimately contribute to pain persistence and disability by limiting movement and reinforcing pain-related beliefs. The avoidance-endurance model (49) further differentiates two maladaptive responses: fear-avoidance behaviors (e.g., movement avoidance due to pain-related fear) can lead to deconditioning and functional decline, while endurance responses (e.g., overactivity despite pain, suppression of pain-related thoughts) may cause tissue overuse and microtrauma. Athletes, who often persist in training despite pain, may be particularly prone to adopt such endurance responses, especially under performance pressure. This highlights the importance of identifying whether psychological and physical alterations in athletes with CPLBP are responses to persistent pain, its cause or the combination of both.

### Strengths and limitations

This scoping review examines the physical, and psychological aspects associated with CPLBP in athletes, offering a more comprehensive understanding of the complexity of this condition by considering multiple dimensions of athletes' health. Additionally, the inclusion of various assessment methods, such as EMG, kinetic and kinematic systems, as well as psychological self-reported outcomes, allows for the capture of a full range of variables relevant to the study of CPLBP in athletes. By focusing on trained athletes, this review addresses the urgent need for studies specific to this athletic population, where CPLBP can have significant impacts on athletic careers.

However, only 11 studies were included, which limits the scope of the conclusions and may not capture the full variability of results in this field of research. The language of the studies was restricted to English and French, which further limited the selection of studies. Because five studies were excluded due to language restrictions, it is possible that some relevant information was missed. However, it is unlikely that the main trends and conclusions of the study have been significantly affected. Among the studies included in this scoping review, a lack of standardization and rigour in the inclusion and exclusion criteria was frequently observed. Generally, the criteria used to determine whether a participant is considered an athlete are not well-defined. Therefore, the criteria used in this scoping review, was based on a definition of a trained athlete from a study that classified athlete levels, allowing us to formulate our own stricter criteria (25). Approximately 37% of the athletes studied were women, highlighting a lack of data for this portion of the population. Additionally, studies that included both men and women did not compare them, except for the study by Osuka et al. (2024) (30). In some cases, the sample sizes were very small, which may have constrained significant observations and conclusions.

The diversity of interventions and measurement tools used in the included studies may complicate the comparability of results and the generalization of conclusions. Most evaluations in these studies involve simple tasks (e.g., jumping, strength, flexion), but more complex movements are necessary to better represent tasks relevant to the athletes' activities. Sheikhhoseini et al. (2016) (46) emphasize the importance of selecting the most pain-provoking movements during sports to conduct these studies. Additionally, there is notable diversity in the types of sports studied, even within individual studies. While this approach provides data and insights on a broader population of athletes, the protocols are not adapted to specific sports, which could introduce bias into the findings. This wide variability in both sports and protocols may explain the significant differences in the results.

## Conclusion

This scoping review has highlighted the complexities associated with CPLBP in athletes. The findings indicate significant differences in the physiological and psychological aspects between athletes with CPLBP and those without CPLBP. These observations emphasize the importance of adopting a multidimensional approach to understand and manage this condition. Although progress has been made in CPLBP research, gaps remain in the literature, particularly concerning the standardization of inclusion criteria and study protocols. Further research is needed to explore whether differences exist between trained athletes with and without CPLBP under conditions of fatigue or concerning parameters associated with sports performance. A better understanding of CPLBP in athletes can lead to more effective rehabilitation strategies and return-to-sport programs, promoting healthier and more sustainable athletic careers.

### Perspectives

This scoping review illustrates the links between CPLBP and physical and psychological characteristics. It is important to recognize that CPLBP should be considered within a biopsychosocial framework (7). Thus, future studies should also address social factors, which are particularly important in athletes with LBP (50). Moreover, to better understand the impact of CPLBP in athletes, studies should investigate complex, sport-specific movements under varying conditions of effort and fatigue. Such approach would further clarify how CPLBP impacts athletic performance and guide the development of rehabilitation strategies that target these specific impairments. In addition, research should assess how CPLBP management can impact not only immediate physical performance but also long-term factors like return-to-sport outcomes and career sustainability. More attention should be given to psychological interventions, as the connection between psychological factors and CPLBP in athletes is still not well understood.
