# Supplementary material for: Physical and psychological differences between athletes with and without chronic primary low back pain: a scoping review
Source: Front Sports Act Living. 2025 Aug 4;7:1621796. doi: 10.3389/fspor.2025.1621796 (PMC12360265; doi:10.3389/fspor.2025.1621796)
Supplement: Supplementary file 1 [file Supplementaryfile1.docx]

**Medline**

Recherche non-MeSH

|  |  |  |
| --- | --- | --- |
| 1 | AB ( (low* N3 ("back pain*" OR "back ache*" OR "backache*" OR "back injur*")) ) OR ( ((Lumbal OR lumbar) N2 (pain* OR ache* OR syndrome OR strain* OR injur*)) ) OR ( (Lumbago OR lumbodynia OR lumbalgesia OR lumbalgia) ) OR TI ( (low* N3 ("back pain*" OR "back ache*" OR "backache*" OR "back injur*")) ) OR ( ((Lumbal OR lumbar) N2 (pain* OR ache* OR syndrome OR strain* OR injur*)) ) OR ( (Lumbago OR lumbodynia OR lumbalgesia OR lumbalgia) ) | 47,359 |
| 2 | AB ( (electromyogra* OR kinematic* OR movement* OR biomechanic* OR "motor control" OR "physiologic adaptation" OR neurophysiologi* OR *psycho* OR "Coping strateg*" OR Adaptation OR Adjustment OR "coping behavio*" OR "adaptive behavior*" OR "Psychological adaptation" OR catastroph* OR "pain catastrophizing" OR kinesiophobia OR behavior OR Mindfulness OR acceptation OR "anxiety and depression" OR distraction OR anticipation OR stress OR "mental stress" OR "psychological stress" OR "psychological load" OR "mental load" OR "recovery stress" OR "recovery-stress balance" OR "endurance-related behavior" OR "distress endurance" OR "pain response" OR "mental fatigue" OR resilience) ) OR TI ( (electromyogra* OR kinematic* OR movement* OR biomechanic* OR "motor control" OR "physiologic adaptation" OR neurophysiologi* OR *psycho* OR "Coping strateg*" OR Adaptation OR Adjustment OR "coping behavio*" OR "adaptive behavior*" OR "Psychological adaptation" OR catastroph* OR "pain catastrophizing" OR kinesiophobia OR behavior OR Mindfulness OR acceptation OR "anxiety and depression" OR distraction OR anticipation OR stress OR "mental stress" OR "psychological stress" OR "psychological load" OR "mental load" OR "recovery stress" OR "recovery-stress balance" OR "endurance-related behavior" OR "distress endurance" OR "pain response" OR "mental fatigue" OR resilience) ) | 3, 603, 270 |
|  |  |  |
| 3 | AB ( (Athlete* OR "Sports participant*" OR "Competitive athlete*" OR "High-performance athlete*" OR "Professional athlete*" OR "Competitive sports performer*" OR "Sports competitor*" OR "Athletic Performance*") ) OR ( (Rowing OR rower* OR sculling OR athlet* OR gymnast* OR cricket OR bowler* OR pitcher* OR wrestl* OR hockey OR baseball OR golf OR kayak* OR canoei* OR "hammer throw*" OR "martial art*" OR basketball OR soccer OR football OR *cycling OR running OR swimming) ) OR TI ( (Athlete* OR "Sports participant*" OR "Competitive athlete*" OR "High-performance athlete*" OR "Professional athlete*" OR "Competitive sports performer*" OR "Sports competitor*" OR "Athletic Performance*") ) OR ( (Rowing OR rower* OR sculling OR athlet* OR gymnast* OR cricket OR bowler* OR pitcher* OR wrestl* OR hockey OR baseball OR golf OR kayak* OR canoei* OR "hammer throw*" OR "martial art*" OR basketball OR soccer OR football OR *cycling OR running OR swimming) ) | 346,233 |
|  | S1 AND S2 AND S3 | 616 |

Recherche MeSH

|  |  |  |
| --- | --- | --- |
| 1 | (AB (“Low back pain” OR “Back pain”) OR TI (“Low back bain” OR “Back pain”)) | 60,561 |
| 2 | (AB (“Biomechanical Phenomena" OR "Electromygraphy" OR "Adaptation, Physiological" OR "Neurophysiology" OR "Psychophysiology" OR "Motivation" OR "Catastrophization" OR "Adaptation, Psychological" OR "Models, Biopsychosocial" OR "Self efficacy" OR "Personal autonomy" OR "Anxiety" OR "Depression" OR "social behaviour" OR "Quality of life" OR "psychology" OR "Movement") OR TI (“Biomechanical Phenomena" OR "Electromygraphy" OR "Adaptation, Physiological" OR "Neurophysiology" OR "Psychophysiology" OR "Motivation" OR "Catastrophization" OR "Adaptation, Psychological" OR "Models, Biopsychosocial" OR "Self efficacy" OR "Personal autonomy" OR "Anxiety" OR "Depression" OR "social behaviour" OR "Quality of life" OR "psychology" OR "Movement")) | 1,326,242 |
| 3 | (AB (“Athletes” OR “Athletic performance” OR “Sports”) OR TI (“Athletes” OR “Athletic performance” OR “Sports”)) | 112,738 |
|  | S1 AND S2 AND S3 | 149 |
|  | Mesh OR non Mesh | 719 |

**Cinahl**

Recherche non-MeSH

|  |  |  |
| --- | --- | --- |
| 1 | AB ( (low* N3 ("back pain*" OR "back ache*" OR "backache*" OR "back injur*")) ) OR ( ((Lumbal OR lumbar) N2 (pain* OR ache* OR syndrome OR strain* OR injur*)) ) OR ( (Lumbago OR lumbodynia OR lumbalgesia OR lumbalgia) ) OR TI ( (low* N3 ("back pain*" OR "back ache*" OR "backache*" OR "back injur*")) ) OR ( ((Lumbal OR lumbar) N2 (pain* OR ache* OR syndrome OR strain* OR injur*)) ) OR ( (Lumbago OR lumbodynia OR lumbalgesia OR lumbalgia) ) | 29,096 |
| 2 | AB ( (electromyogra* OR kinematic* OR movement* OR biomechanic* OR "motor control" OR "physiologic adaptation" OR neurophysiologi* OR *psycho* OR "Coping strateg*" OR Adaptation OR Adjustment OR "coping behavio*" OR "adaptive behavior*" OR "Psychological adaptation" OR catastroph* OR "pain catastrophizing" OR kinesiophobia OR behavior OR Mindfulness OR acceptation OR "anxiety and depression" OR distraction OR anticipation OR stress OR "mental stress" OR "psychological stress" OR "psychological load" OR "mental load" OR "recovery stress" OR "recovery-stress balance" OR "endurance-related behavior" OR "distress endurance" OR "pain response" OR "mental fatigue" OR resilience) ) OR TI ( (electromyogra* OR kinematic* OR movement* OR biomechanic* OR "motor control" OR "physiologic adaptation" OR neurophysiologi* OR *psycho* OR "Coping strateg*" OR Adaptation OR Adjustment OR "coping behavio*" OR "adaptive behavior*" OR "Psychological adaptation" OR catastroph* OR "pain catastrophizing" OR kinesiophobia OR behavior OR Mindfulness OR acceptation OR "anxiety and depression" OR distraction OR anticipation OR stress OR "mental stress" OR "psychological stress" OR "psychological load" OR "mental load" OR "recovery stress" OR "recovery-stress balance" OR "endurance-related behavior" OR "distress endurance" OR "pain response" OR "mental fatigue" OR resilience) ) | 912,364 |
|  |  |  |
| 3 | AB ( (Athlete* OR "Sports participant*" OR "Competitive athlete*" OR "High-performance athlete*" OR "Professional athlete*" OR "Competitive sports performer*" OR "Sports competitor*" OR "Athletic Performance*") ) OR ( (Rowing OR rower* OR sculling OR athlet* OR gymnast* OR cricket OR bowler* OR pitcher* OR wrestl* OR hockey OR baseball OR golf OR kayak* OR canoei* OR "hammer throw*" OR "martial art*" OR basketball OR soccer OR football OR *cycling OR running OR swimming) ) OR TI ( (Athlete* OR "Sports participant*" OR "Competitive athlete*" OR "High-performance athlete*" OR "Professional athlete*" OR "Competitive sports performer*" OR "Sports competitor*" OR "Athletic Performance*") ) OR ( (Rowing OR rower* OR sculling OR athlet* OR gymnast* OR cricket OR bowler* OR pitcher* OR wrestl* OR hockey OR baseball OR golf OR kayak* OR canoei* OR "hammer throw*" OR "martial art*" OR basketball OR soccer OR football OR *cycling OR running OR swimming) ) | 122,809 |
|  | S1 AND S2 AND S3 | 419 |

Recherche MeSH

|  |  |  |
| --- | --- | --- |
| 1 | (AB (“Low back pain” OR “Back pain”) OR TI (“Low back bain” OR “Back pain”)) | 30,838 |
| 2 | (AB (“Biomechanics" OR "Electromygraphy" OR "Adaptation, Physiological" OR "Neurophysiology" OR "Psychophysiology" OR "Motivation" OR "Catastrophization" OR "Adaptation, Psychological" OR "Models, Biopsychosocial" OR "Self-efficacy" OR "Anxiety" OR "Depression" OR "social behaviour" OR "Quality of life" OR "psychology" OR "Kinematics" OR "Movement") OR TI (“Biomechanical Phenomena" OR "Electromygraphy" OR "Adaptation, Physiological" OR "Neurophysiology" OR "Psychophysiology" OR "Motivation" OR "Catastrophization" OR "Adaptation, Psychological" OR "Models, Biopsychosocial" OR "Self-efficacy" OR "Anxiety" OR "Depression" OR "social behaviour" OR "Quality of life" OR "psychology" OR "Kinematics" OR "Movement")) | 478,131 |
| 3 | (AB (“Athletes” OR “Athletic performance” OR “Sports”) OR TI (“Athletes” OR “Athletic performance” OR “Sports”)) | 62,755 |
|  | S1 AND S2 AND S3 | 102 |
|  | Mesh OR non Mesh | 477 |

**Sport Discus**

Recherche non-MeSH

|  |  |  |
| --- | --- | --- |
| 1 | AB ( (low* N3 ("back pain*" OR "back ache*" OR "backache*" OR "back injur*")) ) OR ( ((Lumbal OR lumbar) N2 (pain* OR ache* OR syndrome OR strain* OR injur*)) ) OR ( (Lumbago OR lumbodynia OR lumbalgesia OR lumbalgia) ) OR TI ( (low* N3 ("back pain*" OR "back ache*" OR "backache*" OR "back injur*")) ) OR ( ((Lumbal OR lumbar) N2 (pain* OR ache* OR syndrome OR strain* OR injur*)) ) OR ( (Lumbago OR lumbodynia OR lumbalgesia OR lumbalgia) ) | 9,124 |
| 2 | AB ( (Athlete* OR "Sports participant*" OR "Competitive athlete*" OR "High-performance athlete*" OR "Professional athlete*" OR "Competitive sports performer*" OR "Sports competitor*" OR "Athletic Performance*") ) OR ( (Rowing OR rower* OR sculling OR athlet* OR gymnast* OR cricket OR bowler* OR pitcher* OR wrestl* OR hockey OR baseball OR golf OR kayak* OR canoei* OR "hammer throw*" OR "martial art*" OR basketball OR soccer OR football OR *cycling OR running OR swimming) ) OR TI ( (Athlete* OR "Sports participant*" OR "Competitive athlete*" OR "High-performance athlete*" OR "Professional athlete*" OR "Competitive sports performer*" OR "Sports competitor*" OR "Athletic Performance*") ) OR ( (Rowing OR rower* OR sculling OR athlet* OR gymnast* OR cricket OR bowler* OR pitcher* OR wrestl* OR hockey OR baseball OR golf OR kayak* OR canoei* OR "hammer throw*" OR "martial art*" OR basketball OR soccer OR football OR *cycling OR running OR swimming) ) | 1,053,389 |
| 3 | AB ( (electromyogra* OR kinematic* OR movement* OR biomechanic* OR "motor control" OR "physiologic adaptation" OR neurophysiologi* OR *psycho* OR "Coping strateg*" OR Adaptation OR Adjustment OR "coping behavio*" OR "adaptive behavior*" OR "Psychological adaptation" OR catastroph* OR "pain catastrophizing" OR kinesiophobia OR behavior OR Mindfulness OR acceptation OR "anxiety and depression" OR distraction OR anticipation OR stress OR "mental stress" OR "psychological stress" OR "psychological load" OR "mental load" OR "recovery stress" OR "recovery-stress balance" OR "endurance-related behavior" OR "distress endurance" OR "pain response" OR "mental fatigue" OR resilience) ) OR TI ( (electromyogra* OR kinematic* OR movement* OR biomechanic* OR "motor control" OR "physiologic adaptation" OR neurophysiologi* OR *psycho* OR "Coping strateg*" OR Adaptation OR Adjustment OR "coping behavio*" OR "adaptive behavior*" OR "Psychological adaptation" OR catastroph* OR "pain catastrophizing" OR kinesiophobia OR behavior OR Mindfulness OR acceptation OR "anxiety and depression" OR distraction OR anticipation OR stress OR "mental stress" OR "psychological stress" OR "psychological load" OR "mental load" OR "recovery stress" OR "recovery-stress balance" OR "endurance-related behavior" OR "distress endurance" OR "pain response" OR "mental fatigue" OR resilience) ) | 287,785 |
|  | S1 AND S2 AND S3 | 488 |

Recherche MeSH / SU

|  |  |  |
| --- | --- | --- |
| 1 | (AB (“Low back pain” OR “Back pain”) OR TI (“Low back bain” OR “Back pain”)) | 10,075 |
| 2 | (AB (“Biomechanics" OR "Electromygraphy" OR "Neurophysiology" OR "Psychophysiology" OR "Self-efficacy" OR "Anxiety" OR "Mental Depression" OR "Social conditions of athletes" OR "Quality of life" OR "psychology" OR "Kinematics") OR TI (“Biomechanics" OR "Electromygraphy" OR "Neurophysiology" OR "Psychophysiology" OR "Self-efficacy" OR "Anxiety" OR "Mental Depression" OR "Social conditions of athletes" OR "Quality of life" OR "psychology" OR "Kinematics")) | 70,527 |
| 3 | (AB (“Athletes” OR “Sports”) OR TI (“Athletes” OR “Sports”)) | 278,578 |
|  | S1 AND S2 AND S3 | 73 |
|  | Mesh OR non Mesh | 522 |

**Psychinfo**

Recherche non-MeSH

|  |  |  |
| --- | --- | --- |
| 1 | AB ( (low* N3 ("back pain*" OR "back ache*" OR "backache*" OR "back injur*")) ) OR ( ((Lumbal OR lumbar) N2 (pain* OR ache* OR syndrome OR strain* OR injur*)) ) OR ( (Lumbago OR lumbodynia OR lumbalgesia OR lumbalgia) ) OR TI ( (low* N3 ("back pain*" OR "back ache*" OR "backache*" OR "back injur*")) ) OR ( ((Lumbal OR lumbar) N2 (pain* OR ache* OR syndrome OR strain* OR injur*)) ) OR ( (Lumbago OR lumbodynia OR lumbalgesia OR lumbalgia) ) | 4,994 |
| 2 | AB ( (electromyogra* OR kinematic* OR movement* OR biomechanic* OR "motor control" OR "physiologic adaptation" OR neurophysiologi* OR *psycho* OR "Coping strateg*" OR Adaptation OR Adjustment OR "coping behavio*" OR "adaptive behavior*" OR "Psychological adaptation" OR catastroph* OR "pain catastrophizing" OR kinesiophobia OR behavior OR Mindfulness OR acceptation OR "anxiety and depression" OR distraction OR anticipation OR stress OR "mental stress" OR "psychological stress" OR "psychological load" OR "mental load" OR "recovery stress" OR "recovery-stress balance" OR "endurance-related behavior" OR "distress endurance" OR "pain response" OR "mental fatigue" OR resilience) ) OR TI ( (electromyogra* OR kinematic* OR movement* OR biomechanic* OR "motor control" OR "physiologic adaptation" OR neurophysiologi* OR *psycho* OR "Coping strateg*" OR Adaptation OR Adjustment OR "coping behavio*" OR "adaptive behavior*" OR "Psychological adaptation" OR catastroph* OR "pain catastrophizing" OR kinesiophobia OR behavior OR Mindfulness OR acceptation OR "anxiety and depression" OR distraction OR anticipation OR stress OR "mental stress" OR "psychological stress" OR "psychological load" OR "mental load" OR "recovery stress" OR "recovery-stress balance" OR "endurance-related behavior" OR "distress endurance" OR "pain response" OR "mental fatigue" OR resilience) ) | 2,256,086 |
|  |  |  |
| 3 | AB ( (Athlete* OR "Sports participant*" OR "Competitive athlete*" OR "High-performance athlete*" OR "Professional athlete*" OR "Competitive sports performer*" OR "Sports competitor*" OR "Athletic Performance*") ) OR ( (Rowing OR rower* OR sculling OR athlet* OR gymnast* OR cricket OR bowler* OR pitcher* OR wrestl* OR hockey OR baseball OR golf OR kayak* OR canoei* OR "hammer throw*" OR "martial art*" OR basketball OR soccer OR football OR *cycling OR running OR swimming) ) OR TI ( (Athlete* OR "Sports participant*" OR "Competitive athlete*" OR "High-performance athlete*" OR "Professional athlete*" OR "Competitive sports performer*" OR "Sports competitor*" OR "Athletic Performance*") ) OR ( (Rowing OR rower* OR sculling OR athlet* OR gymnast* OR cricket OR bowler* OR pitcher* OR wrestl* OR hockey OR baseball OR golf OR kayak* OR canoei* OR "hammer throw*" OR "martial art*" OR basketball OR soccer OR football OR *cycling OR running OR swimming) ) | 81,172 |
|  | S1 AND S2 AND S3 | 43 |

Recherche MeSH

|  |  |  |
| --- | --- | --- |
| 1 | (AB (“Back pain”) OR TI (“Back pain”)) | 6,610 |
| 2 | (AB (“Biomechanics" OR "Electromygraphy" OR "motor control" OR "Neurophysiology" OR "Psychophysiology" OR "Motivation" OR "Catastrophizing" OR "Biopsychosocial Approach" OR "Anxiety" OR "Depression (Emotion)" OR "social behaviour" OR "Quality of life" OR "psychology") OR TI (“Biomechanics" OR "Electromygraphy" OR "motor control" OR "Neurophysiology" OR "Psychophysiology" OR "Motivation" OR "Catastrophizing" OR "Biopsychosocial Approach" OR "Anxiety" OR "Depression (Emotion)" OR "social behaviour" OR "Quality of life" OR "psychology")) | 676,240 |
| 3 | (AB (“Athletes” OR “Athletic performance” OR “Sports”) OR TI (“Athletes” OR “Athletic performance” OR “Sports”)) | 36,576 |
|  | S1 AND S2 AND S3 | 8 |
|  | Mesh OR non Mesh | 46 |

| Medline (Msh OR NonMsh) | 719 |
| --- | --- |
| Cinahl (Msh OR NonMsh) | 477 |
| SportDiscuss (Msh OR NonMsh) | 522 |
| Psycinfo | 46 |
| TOTAL | 1764 |
