# Supplementary material for: Physical and psychological differences between athletes with and without chronic primary low back pain: a scoping review
Source: Front Sports Act Living. 2025 Aug 4;7:1621796. doi: 10.3389/fspor.2025.1621796 (PMC12360265; doi:10.3389/fspor.2025.1621796)
Supplement: Supplementary file 2 [file Supplementaryfile2.docx]

| **Authors, date (COUNTRY)** | **Aim / purpose of study: study design** | **Population, Age (SD): Pathology** | **Sport and Level of expertise** | **Evaluation** | **Results** | **Tools and questionnaires to compare athletes with and without CPLBP** |
| --- | --- | --- | --- | --- | --- | --- |
| E. Alijanpour; A. Abbasi; R. A. Needham; R. Naemi, 2021 (IRAN) | Compare the spine-pelvis coordination and coordination variability during rowing in elite rowers with and without CLBP: Cross-sectional study | A = 8 (4W and 4M), 24.12 (4.90): CLBP | Rowing: members of the national rowing team | Endurance: Incremental step-test | The lower back range of motion in transverse plane at 100% intensity was significantly lower in healthy group compared to CLBP group. (table 2) | **Kinematic**: Vero 2.2, VICON, Oxford, UK |
|  |  | A = 6 (3W and 3M), 25.03 (4.50): Healthy |  |  | Coordination: the results of Lower Trunk/ Lower Back showed that into in-phase with proximal dominancy frequency in sagittal plane significantly decreased in CLBP compared to healthy rowers at both intensities for the boat roll-out, blade extraction, and early recovery phases (Fig 4) |  |
|  |  |  |  |  | Coordination variability: the results of post-hoc did not show any significant differences between groups. |  |
| Z. Amerian; S. ShahAli; Z. S. Rezaeian; S. Shanbehzadeh, 2023 (IRAN) | Compare the dynamic postural control between women athletes with and without NSLBP with high and low pain related anxiety: Case-control study | A = 15W, 22.93 (3.53): NSPLBP with High  pain-related  anxiety | Jumping, cutting, and pivoting sports like handball or basketball) or sports requiring less jumping or hard cutting like volleyball, or gymnastics: All participants were self-reported collegiate-level athletes being active competitively, defined as exercising at least 6 h a week | Strength and explosivity: double-leg vertical drop jump and single leg vertical jump | No significant difference between groups in all TTS parameters during SVJ. There was a significant difference between groups for the TTS measures during DVJ. anteroposterior and vertical TTS, the time taken to recover stability was longer for the NSLBP group with high pain-related anxiety compared to both the NSLBP group with low pain related anxiety and the control groups. Only the TTS in the anteroposterior direction was longer in the group of NSLBP with low pain-related anxiety compared to the control group. (Table 2) | **Force plate:** Kistler force plate (type 9260AA6, Kistler Instruments Inc, Switzerland) AND **State-Trait Anxiety Inventory questionnaire** and **Pain-related anxiety** |
|  |  | A = 15W, 22.86 (2.89): NSPLBP with Low pain-related  anxiety |  |  | Significant differences between groups were observed only for total RMS distance during the DJV task. Post hoc analysis revealed greater total RMS distance in the control group compared to both NSLBP groups with high and low pain-related anxiety. None of the COP parameters were different between the groups during SVJ (Table 2) |  |
|  |  | A = 15W, 23.80 (5.46): Healthy |  |  |  |  |
| A. F. Burnett; M. W. Cornelius; W. Dankaerts; P. B. O'Sullivan, 2004 (AUSTRALIA) | Examine whether differences existed in spinal kinematics and trunk muscle activity in cyclists with and without NSCLBP: pilot study | A = 9 (5W and 4M), 42.3 (9.7): NSCLBP | Middle level to high level cyclists/triathletes, | Endurance: Cycling to exhaustion | No significant differences in the lumbar and thoracic flexion angle. (Table 2, 3) | **EMG**: Bortec Electronics, Ont., Canada. Spinal **kinematic**: 3-Space Fastrak, Polhemus Navigation Sciences Division, Vermont, USA. |
|  |  | A = 9 (5W and 4M), 37.6 (7.9): Healthy |  |  | No significant differences in trunk muscle activity. (Table 4, 5, 6) |  |
| E. Marineau Belanger; D.-M. Boon; M. Descarreaux; J. Abboud, 2022 (CANADA) | Assess the neuromuscular control by considering trunk neuromuscular activity and kinematic during constant and controlled cycling effort in healthy cyclists versus cyclists with CLBP: Cross-sectional study | A = 21 (8W and 13M), 43.7 (14.4): NSCLBP | Being able to cycle for at least one hour; having been cycling more than 1000 km per year for more than 2 consecutive years. | Endurance: One-hour cycling | Kinematic: significant group effect was only observed for the  angle 3 (thoracic). (Fig 3) | **Smart trainer**, which measured their pedalling cadence and power output (Kickr, Wahoo, Atlanta, USA). **Bipolar EMG**: Model DE2.1, Delsys Inc., Boston, MA, USA. **Kinematic:** Optotrak Certus, Northern Digital, Waterloo, ON, Canada. AND **VAS** to assess back pain during cycling effort for athletes with and without CPLBP |
|  |  | A = 27 (9W and 18M), 35.0 (13.3): Healthy |  |  | EMG: No difference between group in trunk muscle activity. (Fig 4) |  |
|  |  |  |  |  | MVC: No difference between group in trunk extension strength |  |
| M. Moreno Catalá; A. Schroll; G. Laube; A. Arampatzis, 2018 (GERMANY) | Investigate the athletic-based specificity of muscle strength and neuromuscular control of spine stability in NSCLBP, performing a systematic comparison between athletes and non-athletes with and without NSCLBP: Cross-sectional observational | A = 15 (5W and 10M), 23(2): NSCLBP | soccer, handball, judo, gymnastics, and athletics (discus and javelin throwing): trained at least four times a week and participated regularly in national or international competitions. | Trunk perturbation and functional task: lifting test | Significant pain effect on the maximum isometric trunk extension moments in all three investigated trunk positions, indicating a lower extension strength in LBP athletes compared to healthy athletes (Table 2) | **Dynamometer:** Biodex 3 Medical System Inc., United States. **Force sensor:** MEGATRON Elektronik GmbH & Co. KG, Germany, 0–5 kN, 2073 Hz. **Kinematic**: Vicon Motion Systems, United Kingdom, 250 Hz. **Bipolar EMG:** Biovision GmbH, Germany |
|  |  | NA = 15 (5W and 10M), 27(1): NSCLBP |  |  |  |  |
|  |  | A = 15 (5W and 10M), 23(3): Healthy |  |  | Trunk damping was significantly higher for the LBP participants. Significant pain effect on the muscle onset times of the lumbar and thoracic erector spinae muscles, evidencing shorter muscle reaction times after release in the LBP athletes. (Table 3) |  |
|  |  | NA = 14 (5W and 9M), 24(3): Healthy |  |  |  |  |
| M. Movahed; M. Salavati; R. Sheikhhoseini; A. M. Arab; K. O'Sullivan, 2019 (IRAN) | Compare kinematics of the lower extremity and lumbar spine during a single leg landing task between female volleyball athletes with and without persistent LBP: Cross-sectional study | A = 18W, 21.78 (2.65): NSCLBP | Playing volleyball three times weekly for at least three years | Single leg landing task | The LBP group had significantly more lumbar extension at initial contact and at the time of maximal vertical ground reaction force with no other significant kinematic differences observed between the groups. (Table 3) | **Kinematic**: Vicon MX, Oxford Metrics, UK. **Force plate**: Kistler, Winterthur, Switzerland |
|  |  | A = 18W, 20.78 (2.42): Healthy |  |  |  |  |
| S. Osuka; Y. Koshino; K. Watanabe; Y. Kataoka; H. Tohyama, 2024 (JAPAN) | Determine the psychosocial factors associated with NSCLBP among college athletes: Cross-sectional investigation | A = 41, 20.0 (2.0): NSCLBP | All sports (table 1): athletes belonging to 18  clubs within our university (11h per week) | No physical evaluation | The FABQ-PA, TSK-11, PCS scores in the NS-CLBP group were significantly higher than those of the non-NS-CLBP group (Table 3). No significant difference was found in the AFAQ score. The TSK-11 and RDQ scores showed a small effect size, and the FABQ-PA and PCS scores revealed moderate effect sizes. | **FABQ-PA, AFAQ**, **TSK-11, PCS** |
|  |  | A = 222, 20.0 (2.0): Healthy |  |  |  |  |
| T. Renkawitz; D. Boluki; J. Grifka, 2006 (Germany) | Examine the relationship between LBP, neuromuscular imbalance, clinical symptoms, and trunk extension strength on two independent occasions, after dynamic neuromuscular changes through a back exercise program: Experimental longitudinal study | A = 46 (19W and 27M), 34,1 (7,1): CLBP | Elite amateur tennis players who take part in team competitions and tennis tournaments regularly. | Back flexibility and spinal mobility | Significant neuromuscular imbalances between right and left erector spinae at the L2 and L4 lumbar levels during maximum voluntary trunk extension among subjects with LBP. (Fig 3) | **Surface EMG**: Triode; Thought Technology Ltd., Montreal, Canada |
|  |  | A = 36 (12W and 24M), 31,85 (7,3): Healthy |  |  | trunk extension strength: No difference between athletes with and without CPLBP (Fig 3) |  |
| R. Sheikhhoseini, M-H. Alizadeh, M. Salavati, K. O'Sullivan, E. Shirzad, M. Movahed, 2018 (IRAN) | Examine lumbopelvic and lower extremity kinematics in athletes with persistent LBP during a jumping task: comparative cross-sectional study | A = 20W, 21,45 (2,70): active extension CLBP | Playing volleyball three times weekly for at least three years | Jump-Landing-Jump | Athletes with LBP displayed significantly less knee flexion and significantly greater hip flexion than the group without LBP at the lowest point of the jump-land-jump maneuver. There were no statistically significant differences between the other variables (Table 3) | **Kinematic:** Vicon 6 camera motion capture system (Vicon MX, Oxford Metrics, UK) |
|  |  | A = 18W, 20,78 (2,42): Healthy |  |  |  |  |
| S. Shenoy; Balach; H. er; J. S. hu, 2013 (INDIA) | Compare long latency reflex response (occurring between 40 to ∼ 100 ms duration) in athletes with CLBP with asymptomatic athletes: Cross-sectional study | A = 24 (8W and 16M), 24.26 (4.7): NSCLBP | Soccer, hockey, handball, basketball: playing for a minimum of five days a week for the last three years. | Expected and unexpected perturbations | Unexpected perturbation, the latency of onset for both RA and ES were significantly delayed in the LBP group. (Table 2) | **Surface EMG:** Noraxon-MyoS 1200 set manufactured by  Noraxon (USA) |
|  |  | A = 25 (8W and 17M), 25.13 (5.05): Healthy |  |  | Mean EMG amplitude: The results showed statistically significant difference between LBP and control groups in Expected task of RA and Unexpected tasks of both RA and ES, but no significant result was found in Expected task of ES. (Table 2) |  |
| S. Shenoy; Balach; H. er; J. S. hu, 2014 (INDIA) | Compare the effect of prior instructions on the PPR (preprogrammed muscle reaction) amplitude in the trunk muscles in individuals with CLBP compared to healthy controls: Cross-sectional study | A = 24 (8W and 16M), 24.26 (4.7): NSCLBP | Soccer, hockey, handball, basketball: five days a week for the last three years. | Expected and unexpected perturbations | EMG amplitude: significant difference between LBP and control groups for both commands in the RA muscle group. In the ES, no significant difference was obtained for the command “let go,” while “resist” showed a significant mean difference between the LBP and control groups. (Table 2) | **Surface EMG**: Noraxon-MyoS 1200 set manufactured by  Noraxon (USA) |
|  |  | A = 25 (8W and 17M), 25.13 (5.05): Healthy |  |  |  |  |

Notes : A (Athletes); NA (Non Athletes); LBP (Low Back Pain); CLBP (Chronic Low Back Pain); CPLBP (Chronic Primary Low Back Pain); NSCLBP (Non-Specific Chronic Low Back Pain); NSPLBP (Non-Specific Persistent Low Back Pain); W (Women); M (Men); SD (Standard deviation); TTS (Time To Stabilization); SVJ (single-leg vertical jump); DVJ (double-leg vertical drop jump); RMS (Root Mean Square); COP (Center Of Pressure); EMG (Electromyography); MVC (Maximal Voluntary Contraction); FABQ-PA (FABQ-PA, Fear-Avoidance Beliefs Questionnaire Physical Activity subscale); TSK-11 (Tampa Scale for Kinesiophobia-11); PCS (Pain Catastrophizing Scale); AFAQ (Athlete Fear Avoidance Questionnaire); VAS (Visual Analogue Scale); RA (Rectus Abdominis); ES (Erector Spinae).
